# IgG persistence showed weak clinical aspects in chronic schistosomiasis patients

**DOI:** 10.1038/s41598-023-40082-z

**Published:** 2023-08-14

**Authors:** Shudong Xie, Yu Zhang, Junhui Li, Jie Zhou, Jun Li, Pengpeng Zhang, Yang Liu, Yulin Luo, Yingzi Ming

**Affiliations:** 1 Transplantation Center, The Third Xiangya Hospital, Central South University, No. 138 Tongzipo Road, Changsha, 410013 Hunan People’s Republic of China; 2 Engineering and Technology Research Center for Transplantation Medicine of National Health Comission, Changsha, Hunan People’s Republic of China; 3 Hunan Institute of Schistosomiasis Control, Yueyang, Hunan People’s Republic of China

**Keywords:** Immunology, Microbiology, Biomarkers, Diseases, Medical research

## Abstract

Schistosomiasis is a chronic parasitic disease, which affects the quality of daily life of patients and imposes a huge burden on society. Hepatic fibrosis in response to continuous insult of eggs to the liver is a significant cause of morbidity and mortality. However, the mechanisms of hepatic fibrosis in schistosomiasis are largely undefined. The purpose of our study is to detect the indicator to hepatic fibrosis in schistosomiasis. A total of 488 patients with chronic schistosomiasis japonica were enrolled in our study. The patients were divided into two groups according to liver ultrasound examination, which could indicate liver fibrosis of schistosomiasis with unique reticular changes. Logistic regression analysis showed that globulin, albumin/globulin, GGT levels and anti-*Schistosoma* IgG were independently associated with liver fibrosis in patients with schistosomiasis and IgG was the largest association of liver fibrosis (OR  2.039, 95% CI 1.293–3.213). We further compared IgG+ patients with IgG− patients. IgG+ patients (ALT 25 U/L, GGT 31 U/L) slightly higher than IgG− patients (ALT 22 U/L, GGT 26 U/L) in ALT and GGT. However, the fibrosis of liver in IgG+ patients (Grade II(19.7%), Grade III(7.3%)) were more severe than that in IgG− patients(Grade II(12.5%), Grade III(2.9%)) according to the grade of liver ultrasonography. Our results showed anti-*Schistosoma* IgG was independently associated with liver fibrosis in patients with chronic schistosomiasis japonica and patients with persistent anti-*Schistosoma* IgG might have more liver fibrosis than negative patients despite no obvious clinical signs or symptoms.

## Introduction

Schistosomiasis is a water-borne tropical infectious parasitic disease with an affect more than 200 million people in approximately 78 countries, remaining a major public health concern worldwide^1^. Although the incidence and prevalence of schistosomiasis have decreased, there are still some neglected schistosomiasis patients and sporadic in low-income and middle-income countries in Africa and Asia^2–4^. Human schistosomiasis is mainly caused by *Schistosoma japonicum (S. japonicum)*, *Schistosoma mansoni* and *Schistosoma aegypti* which different from S. haematobium^5^. However, *S. japonicum* is mainly popular in China, Japan, Korea and Southeast Asia^6^. Acute schistosomiasis with fever, cough, diarrhea, anorexia, or arthralgias usually are improved by praziquantel which only kills the adult worms^7,8^. *Schistosoma* eggs are mainly the pathogenic factor of chronic schistosomiasis^7,8^. The eggs parasitizing on host tissues induce chronic immunopathological reactions which lead to collagen-rich granuloma formation and liver fibrosis in *Schistosoma japonica* infection^9,10^. Presinusoidal portal hypertension in advanced schistosomiasis are the main reason for the mortality of schistosomiasis^11,12^. The mechanisms of hepatic fibrosis in schistosomiasis are largely undefined and it is important to note that the rapid and effective identification of early fibrosis is critical to the prognosis of patients with schistosomiasis japonica.

In schistosomiasis, Th1 immune responses at acute stage of infection progress to a Th2 response at egg-stage^13,14^. Chronic schistosomiasis occurs mainly as a result of granulomatous inflammation induced by the parasite eggs trapped in the liver. An influx of immune cells to the liver, particularly macrophages, eosinophils, neutrophils, T-cells and B-cells, leads to form granulomas around egg^10,15^. Following infection, type 1 immune dominated response is elicited. This response is characterized by increased release of Interleukin(IL)-12 and interferon (IFN)-γ and is mainly targeted at worm antigens mainly dominated by macrophages^16^. As the parasite matures and starts to produce eggs, a shift toward a type 2-biased immune response occurs. Type 2 immune response is characterized by expansion of Th2 cells, eosinophils, and basophils, increased production of IL-4, IL-5, and IL-13, an isotype switch toward IgG1 and IgE, as well as polarization of macrophages toward the M2 phenotype^17^. The interaction between immune cells promotes extracellular matrix deposition and liver fibrosis.

There is also a close relationship between B cells and schistosomiasis. In the murine model, B cell–deficient mice develop exacerbated egg pathology during both acute and chronic infection with schistosomiasis^18^. The main functions of B cells are antigen presentation and antibody secretion. Antibody responses are thought to play an important role in *Schistosoma* infections and there are five classes of immunoglobulins including IgA, IgD, IgE, IgG, and IgM. In human infection, protective IgA and IgE levels have been demonstrated against adult worm antigens^19^. In addition, antibody may promote protective effects within the liver through local interactions with macrophages^20^. However, the levels of serum *S. japonicum* heat shock protein 60 (SjHSP60)-specific IgG were positively correlated with the severity of liver pathology after *S. japonicum* infection^21^. Meanwhile, the detection of schistosome antibodies is one of the important diagnostic methods for schistosomiasis. It is interesting that some patients with chronic schistosomiasis are continual presence of anti-*Schistosoma* IgG, while some patients switched back from positive to negative after several years^22,23^. Previous studies have shown that humoral response in parasite susceptibility has been well established, but its roles in clinic outcome remains poorly understood in patients with chronic schistosomiasis japonica^24,25^. Anti-*Schistosoma* antibody might be tested as biomarkers of disease severity.

Liver fibrosis is associated with the development of schistosomiasis and leading cause of death in patients with schistosomiasis^8,9^*.* However, in the chronic phase of schistosomiasis, more than 90% of individuals in early stage of liver fibrosis are asymptomatic or have mild clinical manifestations because of the strong compensation ability of the liver and a few of them develop into severs liver fibrosis, which subsequently undergo portal hypertension, splenomegaly, oesophageal varices and so on^26–28^. Evidently, asymptomatic liver fibrosis is a successful adaptation of host-parasite relation. However, the mechanism of liver fibrosis in schistosomiasis is required for further research and early intervention of liver fibrosis might improve the prognosis of patients. Thus, our aim is to identify potential risk factors and indicators for early liver fibrosis in schistosomiasis and investigate the association between the persistence of anti-*Schistosoma* IgG and schistosome-associated liver fibrosis.

## Results

### General characteristics of the study population

Our study included 488 participants diagnosed with chronic schistosomiasis japonica without decompensated liver cirrhosis, such as ascites, a giant spleen, or gastroesophageal variceal bleeding. 363 of the 488 individuals were male (74.4%). The ages ranged from 7 to 84 years. The mean age was 58.3 years (SD 12.7), and the median was 60 years (IQR 17.5). We retrospectively collected clinical data of the patients, who underwent blood routine, liver function, renal function, ultrasound, and *Schistosoma* IgG testing. In the study population, 387 individuals (79.3%) exhibited liver reticular changes, while 101 individuals (20.7%) showed no liver reticular changes. Among the 488 individuals evaluated in the study, there was no correlation discovered between liver reticular changes and the factors of age and gender (Table 1).

**Table 1:** Characteristics of the study population with or without liver reticular changes (n = 488).

Characteristics	Number (%), N = 488	With liver reticular changes (79.3%), N = 387	Without liver reticular changes (20.7%), N = 101	*P*
Sex				0.1254
Male	363 (74.4)	294 (76.0)	69 (68.3)	
Female	125 (25.6)	93 (24.0)	32 (31.7)	
Age group (years)		58.2 ± 12.9	58 ± 12.1	0.796
7–10	2	0 (0.0)	2 (2)	
11–19	2	2 (0.5)	0 (0)	
20–29	4	3 (0.7)	1 (1)	
30–39	34	31 (8.0)	3 (3)	
40–49	74	54 (14.0)	20 (20)	
50–59	127	105 (27.1)	22 (22)	
60–69	160	110 (28.4)	50 (50)	
70–84	85	81 (20.9)	4 (4)	

### Anti-*Schistosoma* IgG strongly associated with liver reticular changes in patients infected with *S. japonicum*

**Figure 1:**
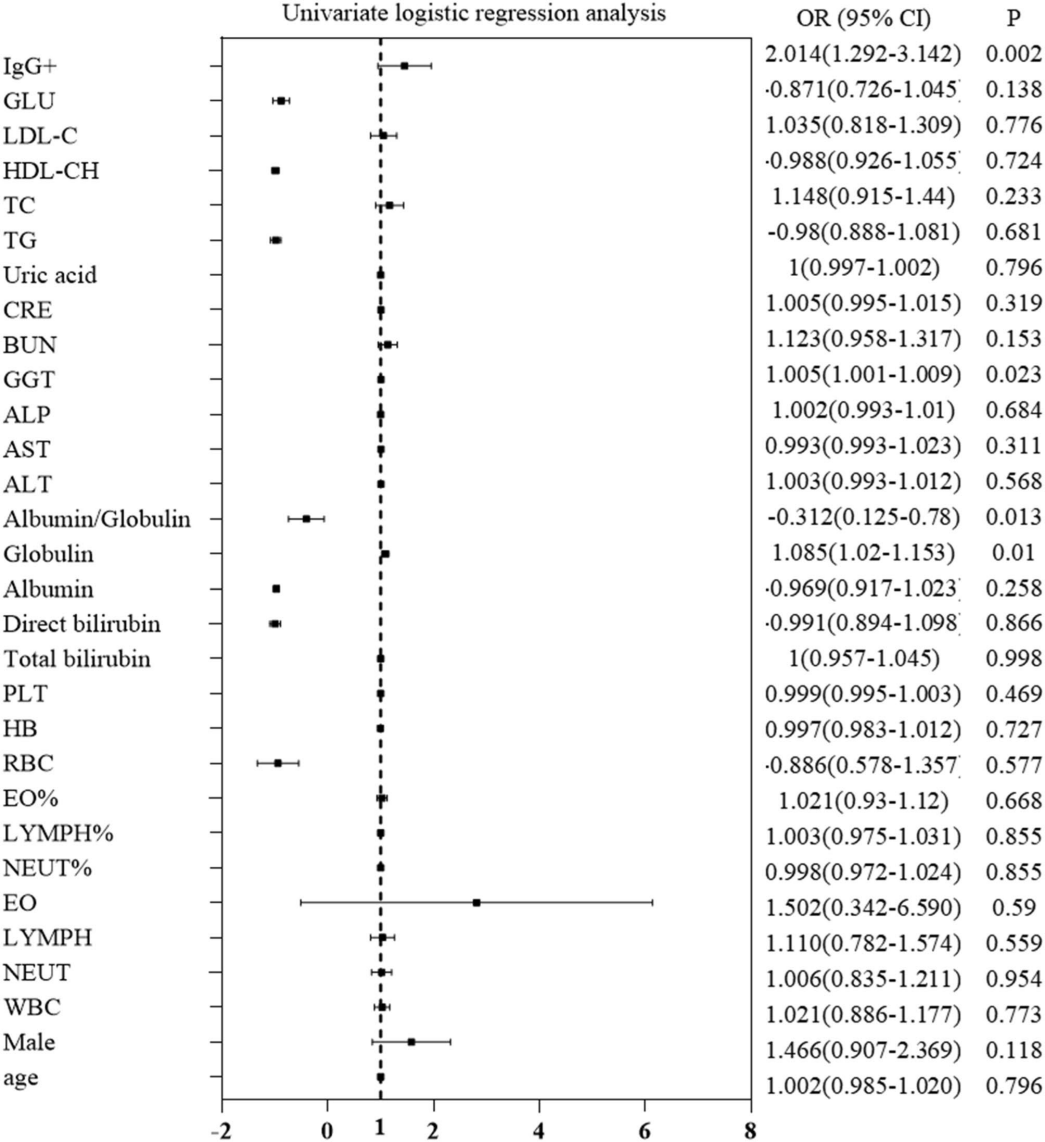
Univariate logistic regression analysis of factors associated with liver reticular changes in chronic patients infected with *S. japonicum*. *WBC* white blood cell count, *NEUT* neutrophil count, *LYMPH* lymphocyte count, *EO* eosinophil count, *RBC* red blood cell count, *HB* hemoglobin, *PLT* platelets, *ALT* alanine aminotransferase, *AST* aspartate aminotransferase, *TB* total bilirubin, *DB* direct bilirubin, *ALB* Albumin, *GLB* globulin, *ALP* alkaline phosphatase, *GGT* glutamyl transpeptidase, *BUN* urea nitrogen, CRE creatinine, *UA* uric acid, *TG* triglycerides, *TC* total cholesterol, *HDL* high-density lipoprotein, *LDL* low-density lipoprotein, *FBG* fasting blood glucose, *IgG* + positive IgG antibody, *OR Odds ratio, CI confidence interval; for other abbreviations*.

**Figure 2:**
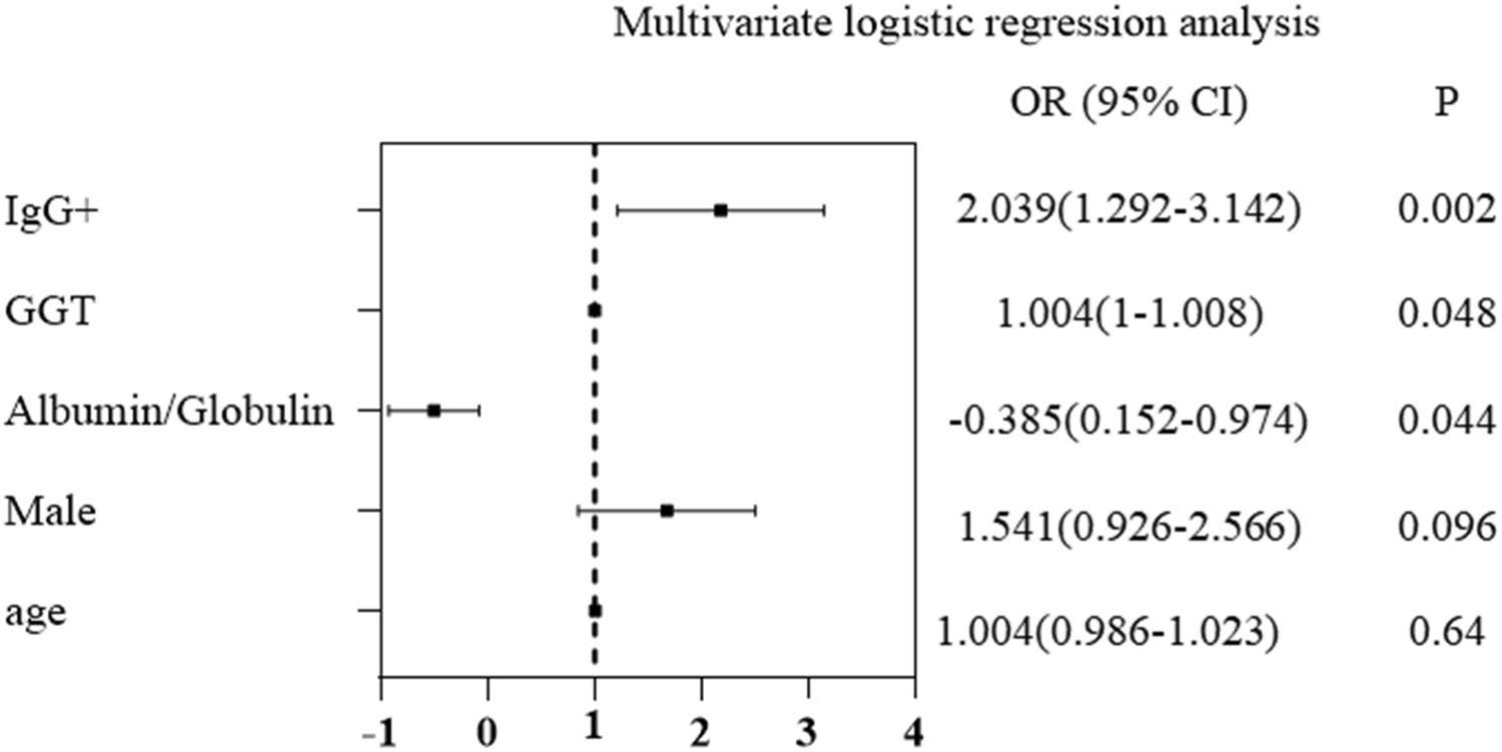
Multivariate logistic regression analysis of factors associated with liver reticular changes in chronic patients infected with *S. japonicum*.

As shown in Fig. 1, univariate logistic regression analysis revealed that Globulin, Albumin/Globulin, GGT levels and anti-*Schistosoma* IgG were associated with liver reticular changes in patients with chronic schistosomiasis japonica. Multivariate logistic regression analysis showed that elevated Albumin/Globulin (adjusted odds ratio (aOR), 95% confidence interval (CI); *P* < 0.05) were inversely associated with liver reticular changes in patients with chronic schistosomiasis japonica. In addition, GGT levels and positive IgG antibody (aOR, 95% CI; *P* < 0.05) were independently associated with a significantly increased risk of liver reticular changes in patients with chronic schistosomiasis japonica (Fig. 2). As IgG had the strongest association with liver reticular changes (OR 2.039, 95% CI  1.293–3.213), general characteristics of the population with or without anti-*Schistosoma* IgG showed in Fig. 2. Age was also not equally distributed throughout the groups where most are concentrated in the age groups of 50 to 59 years and 60 to 69 years. 218(55.3%) of the 488 individuals had positive IgG antibody, 270(44.7%) with negative IgG antibody. Among the 488 individuals evaluated in the groups, there was no association between IgG antibody and age, sex or gender (Table 2).

**Table 2:** Characteristics of the study population with or without IgG (n = 488).

Characteristics	Number (%), N = 488	IgG-(55.3%), N = 270	IgG + (44.7%), N = 218	*P*
Sex				0.702
Male	363 (74.3)	199 (54.8)	164 (45.1)	
Female	125 (25.6)	71 (56.8)	54 (43.2)	
Age group (years)		60 ± 12	56 ± 13	0.224
7–10	2	2 (0.74)	0 (0)	
11–19	2	0 (0)	2 (0.92)	
20–29	4	2 (0.74)	2 (0.92)	
30–39	34	12 (4.44)	22 (10.09)	
40–49	74	34 (12.59)	40 (18.35)	
50–59	127	70 (25.93)	57 (26.15)	
60–69	160	99 (36.67)	61 (27.98)	
70–84	85	51 (18.89)	34 (15.6)	

### Blood routine test, liver and kidney function in patients of chronic schistosomiasis japonica with or without anti-*Schistosoma* IgG

In Table 3, blood routine results were no significantly associated with anti-*Schistosoma* IgG in patients with chronic schistosomiasis japonica. However, PLT had a downward trend in patients with positive IgG antibody (*P* = 0.139) (Table 3). Compared to liver function results of the patients with negative IgG antibody (ALT 22 U/L, GGT 26 U/L), ALT and GGT were significantly higher in the patient with positive IgG antibody (ALT 25 U/L, GGT 31 U/L) (Table 4). DB and AST had a rising trend in patients with positive IgG antibody (DB 3.9 mmol/L, AST 24 U/L) than patients with negative IgG antibody (DB 3.6 mmol/L, AST 23 U/L). As for renal function results, there were not significant difference in chronic schistosomiasis patients with or without positive IgG antibody (Table 4). Although FBG had a downward trend in patients with positive IgG antibody (*P* = 0.068), there were no significant difference between IgG+ patients and IgG- patients about blood sugar and lipids (Table 4).

**Table 3:** The relationship between IgG and blood routine results in patients with chronic schistosomiasis.

Variables	IgG−	IgG+	Z/T	*P*
WBC (*10^9^/L)	5.79 (4.915–6.965)	5.755 (5.01–6.6725)	0.716	0.474
NEUT (*10^9^/L)	3.45 (2.77–4.275)	3.3 (2.76–4.085)	0.858	0.391
LYMPH (*10^9^/L)	1.83 (1.43–2.165)	1.8 (1.4975–2.18)	0.421	0.674
EO (*10^9^/L)	0.14 (0.09–0.24)	0.13 (0.09–0.2125)	1.36	0.174
NEUT (%)	59.376 ± 8.5874	58.52 ± 8.308	− 1.111	0.267
LYMPH (%)	31.1751 ± 8.13463	32.1972 ± 7.55894	1.424	0.155
EO (%)	2.5 (1.5–3.65)	2.3 (1.6–3.5)	0.898	0.369
RBC (*10^12^/L)	4.71 (4.44–5.05)	4.705 (4.3725–5.0225)	0.815	0.415
HB (g/L)	145 (133.5–154)	145 (135–155)	0.265	0.791
PLT (*10^9^/L)	197 (160–240.5)	193 (163.5–224.25)	1.481	0.139

**Table 4:** The relationship between IgG and liver function results, renal function results, blood sugar and lipids in patients with chronic schistosomiasis.

Variables	IgG−	IgG+	Z/T	*P*
TB (mmol/L)	12.4 (10.1–16.15)	12.45 (10.1–17.625)	1.188	0.235
DB (mmol/L)	3.6 (2.85–4.55)	3.9 (2.9–5.1)	1.787	0.074
ALB (g/L)	45.2 (43.5–47)	45.9 (43.35–47.2)	0.807	0.42
GLB (g/L)	25.3 (23.2–27.5)	25.5 (23.3–27.825)	0.767	0.443
ALB/GLB	1.78 (1.635–1.93)	1.78 (1.6175–1.9325)	0.498	0.618
ALT (U/L)	22 (17–31)	25 (18–35)	2.449	0.014
AST (U/L)	23 (19–29)	24 (19–31)	1.878	0.06
ALP (U/L)	67 (54–80)	67 (55–80.25)	0.318	0.75
GGT (U/L)	26 (18–41)	31 (19–48)	2.328	0.02
BUN (mmol/L)	4.5 (3.77–5.56)	4.7 (3.89–5.51)	9.74	0.33
CRE (umol/L)	66.6 (56.1–77.95)	68.5 (57.625–77.225)	0.622	0.534
UA (umol/L)	347 (298.5–405.5)	353.5 (302–406.25)	0.444	0.657
TG (mmol/L)	1.38 (0.97–2.08)	1.505 (1.055–2.1675)	1.627	0.104
TC (mmol/L)	5.17 (4.405–5.725)	4.975 (4.4–5.78)	0.252	0.801
HDL (mmol/L)	1.29 (1.075–1.49)	1.25 (1.0975–1.45)	0.927	0.354
LDL (mmol/L)	3.4496 ± 0.94068	3.4203 ± 0.91769	0.73	0.364
FBG (mmol/L)	5.52 (5–6.185)	5.35 (4.9975–5.75)	1.827	0.068

### Liver fibrosis grade in patients with chronic schistosomiasis japonica with or without anti-*Schistosoma* IgG

Of the total 488 patients received liver ultrasound examination and followed up by professional doctors in a standardized operation process from January 2021 to June 2022. The ratio of positive IgG antibody patients to negative IgG antibody patients was close to 1:1. Among them, 220 patients had liver fibrosis classified as Grade 0, while 167, 77, and 24 patients had liver fibrosis classified as Grades I, II, and III, respectively. The liver fibrosis in IgG+ patients (Grade 0: 41.2%, Grade I: 31.6%, Grade II: 19.7%, Grade III: 7.3%) was found to be more severe compared to IgG- patients (Grade 0: 48.1%, Grade I: 36.2%, Grade II: 12.5%, Grade III: 2.9%) according to the results of liver ultrasonography (Ζ = 2.474, *p* = 0.013) (Table 5). Overall, the rate of liver fibrosis grading II or III was significantly higher in positive IgG patients than those with negative IgG antibody.

**Table 5:** Relationship between IgG and hepatic fibrosis in patients with chronic schistosomiasis.

	Grade 0	Grade I	Grade II	Grade III	Total
IgG−	130 (48.1%)	98 (36.2%)	34 (12.5%)	8 (2.9%)	270
IgG+	90 (41.2%)	69 (31.6%)	43 (19.7%)	16 (7.3%)	218
Subtotal	220	167	77	24	488

## Discussion

Schistosomiasis may remain a significant threat in endemic areas. Liver fibrosis caused by the accumulation of schistosome eggs is a major contributor to death^29^. The mechanism of liver fibrosis is also not fully defined in schistosomiasis japonica. Pathology is the gold standard for detecting liver fibrosis. However, it is difficult to routinely perform biopsies on patients with chronic schistosomiasis. Ultrasonography, being a non-invasive method, is considered to be a complementary tool for diagnosis of schistosomiasis with unique fibrotic pattern^30^. There are no noticeable symptoms or signs before hepatic decompensation occurs. We conducted a retrospective analysis of patients with chronic schistosomiasis japonica to explore the factors and indicators related to liver fibrosis in this condition.

Our study included 488 patients and logistic regression analysis showed that globulin, albumin/globulin, GGT levels and anti-*Schistosoma* IgG were independently associated with liver fibrosis in patients with schistosomiasis. Some studies also showed that globulin and GGT was an risk factor for liver fibrosis in chronic hepatitis B virus (HBV)-infected patients^31,32^. In our study, IgG was the strongest association of liver fibrosis (OR 2.039, 95% CI  1.293–3.213). The role of humoral immunity in chronic *S. japonicum* patients has not yet been completely defined^33^. B cell–deficient mice develop exacerbated egg pathology in schistosomiasis, which indicated B cells might play an important role in hepatic fibrosis of schistosomiasis^18^. There were many studies investigating the relationship between immunoglobulins and schistosomiasis. IgA and IgE have a protective role against adult worm antigens, while IgG was positively associated with severe schistosomiasis^19,21,25,34^. So we further explored the association between IgG to *Schistosoma* and patients with chronic schistosomiasis japonica. All of patients were divided into two group IgG+ patients (n = 218) and IgG− patients (n = 270). We found that ALT, GGT levels and liver fibrosis grades were significantly different in those two groups. IgG+ patients (ALT 25 U/L, GGT 31 U/L) slightly higher than IgG-(ALT 22 U/L, GGT 26 U/L) patients in ALT and GGT. However, the fibrosis of liver in IgG+ patients (Grade 0(41.2%), Grade I (31.6%), Grade II (19.7%), Grade III (7.3%)) were more severe than that in IgG- patients (Grade 0(48.1%), Grade I (36.2%), Grade II (12.5%), Grade III (2.9%)) according to the grade of liver ultrasonography. It is interesting that there is no obvious difference in the most clinical indicators between IgG+ patients and IgG− patients such as routine blood parameters, liver function, kidney function, blood sugar and lipids, thought the grade of liver fibrosis in IgG+ patients is more serious than that in IgG− patients. As the results, we further employed non-invasive methods such as fibrosis-4 index (FIB-4) to better evaluate liver fibrosis in patients. However, there were a discrepancy between FIB-4 and ultrasound in our study. Few patients with high FIB-4 showed mesh-like changes on ultrasound. The FIB-4 is closely correlated with AST and ALT. However, liver is a highly compensatory organ, and liver fibrosis is a chronic and progressive process. In order to identify early indicators of schistosome-associated liver fibrosis, we included chronic schistosomiasis japonica patients with compensated phase of liver. Among the patients we included, only a small number of patients had liver function abnormalities. Therefore, FIB-4 may not be suitable for evaluating liver fibrosis in our study. It might be also the reason why there was no significant difference in various clinical indicators between the IgG+ patients and the IgG− patients, despite the difference in the degree of liver fibrosis.

Our research preliminarily showed that persistence of anti-*Schistosoma* IgG indicated more serious hepatic fibrosis in patients with chronic schistosomiasis japonica. There might be several reasons for the following. Firstly, when the patient's infection were more severe, there was a large number of schistosome eggs in the liver and these eggs secreted a large number of antigens, thereby inducing the body to produce a large number of antibodies and not easy to disappear^35,36^. Secondly, when patients with chronic schistosomiasis japonica were repeatedly infected with schistosome, such as fisherman or boatman in endemic areas, the extent of liver damage in the patient would become more severe, and there might be a continued production of antibodies^37,38^. Meanwhile, we were unaware of both the exact number and times of when the patients became infected. But there was no difference in age between the patients with or without anti-*Schistosoma* IgG, which might minimize the limitations of infection time and number. Deborah also reported that that parasite-reactive IgG levels were associated with signals of disease severity in *Schistosoma mansoni* infected individuals^33^. Parasite-reactive IgG levels were positive associated with parasite burden, longitudinal spleen size, thickness of the portal vein and so on. Chen reported that total IgG were positively correlated with the severity of liver pathology after *S. japonicum* infection in animals (mice and rabbits) or patients^21^. These results were in agreement with our findings. It might be explained by the fact that IgG antibody against *Schistosoma* might play an important role in the process of granuloma formation and liver fibrosis^24,39^. To prevent the development of hepatic fibrosis caused by schistosomiasis, surveillance should be strengthened particularly in those patients who are persistence of anti-*Schistosoma* IgG.

There are several limitations that need to be addressed in our study. First, liver fibrosis was evaluated by colour ultrasound which was in accordance with the World Health Organization diagnostic criteria for *S. japonicum*, lacking of liver biopsies results^24,39^. Second, the reasons affecting IgG antibody changes have not been found. In summary, our study showed anti-*Schistosoma* IgG and elevated Albumin/Globulin and GGT levels were independently associated with liver fibrosis reticular changes in patients with chronic schistosomiasis japonica. In addition, persistence of anti-*Schistosoma* IgG indicated more serious hepatic fibrosis in patients with chronic schistosomiasis japonica and might be potential indicator of disease severity. We will further explore their underlying molecular mechanisms of B cell in liver fibrosis of schistosomiasis.

## Methods

### Study design and population

A medical record review was retrospectively conducted from January 2021 to June 2022 at Xiangyue Hospital, Yueyang City, Hunan Province, China. Yueyang City is located near to Dongting Lake where ecology and environmental factors are conducive to the reproduction of the intermediate host of *S. japonicum*, the snail Oncomelania hupensis. Therefore, this area has historically been a high risk area for schistosomiasis and has many patients infected with schistosomiasis japonica. We included 488 patients of all ages and genders in the study with chronic schistosomiasis who have a history of exposure to contaminated water, positive detection of eggs in their stool, and have received treatment with praziquantel. Praziquantel treatment was administered at 60 mg/kg/day for two consecutive days in chronic* S. japonicum* infected individuals^40,41^. We defined the duration for which anti-*Schistosoma* IgG remains detectable from the time of infection to the inclusion of patients in our study as duration for persistence of IgG. We excluded patients with advanced schistosomiasis who showed symptoms of portal hypertension such as ascites, enlargement of the spleen (splenomegaly), and bleeding from the veins in the esophagus and stomach (gastro-esophageal variceal bleeding)^42^. Meanwhile, patients who tested positive for hepatitis B virus, hepatitis C virus, and human immunodeficiency virus, or who had fatty liver disease caused by alcohol consumption or other factors (as determined by ultrasound scan and alcohol consumption above 30 g daily), decompensated liver disease or liver cancer (as indicated by ultrasound and liver function tests), or had undergone organ transplantation (as self-reported), were also excluded from the study. This study was conducted and approved by the Ethics Committee of the third Xiangya Hospital of Central South University (No: 21149) and has been carried out in accordance with the Code of Ethics of the World Medical Association (Declaration of Helsinki) for experiments. All methods were performed in accordance with the relevant guidelines and regulations. All adult participants and parents of minors provided written informed consent and minors provided assent prior to the study. The privacy of all participants is fully protected.

### Diagnosis of *S. japonicum* infection and liver fibrosis grade

**Figure 3:**
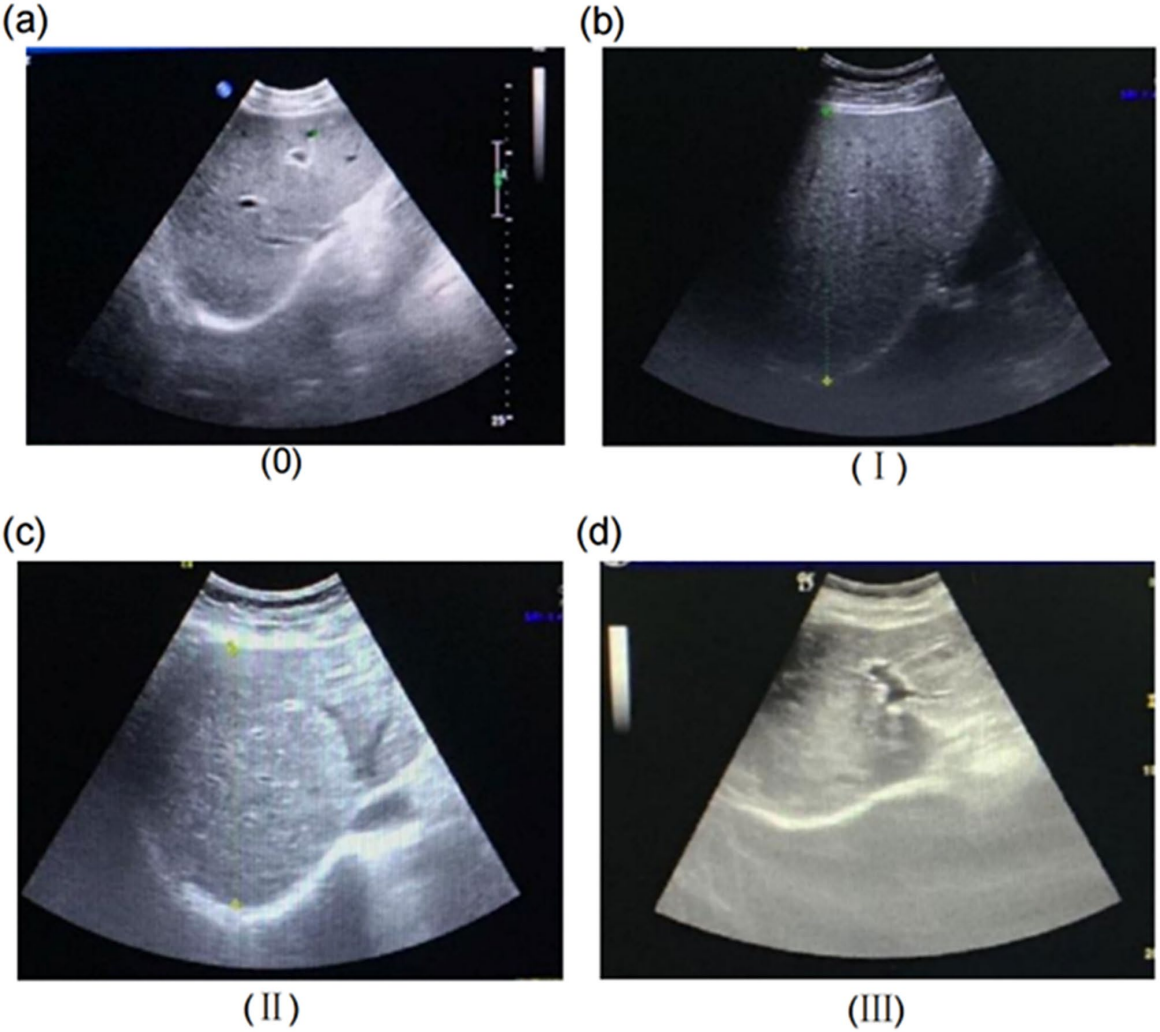
The hepatic fibrosis was graded from 0 to III: (**a**) Grade 0: normal and thicker light spot type, normal liver sonogram or only thick liver parenchyma echo; (**b**) Grade I: focal echoes in the liver parenchyma are scattered without clear boundary; (**c**) Grade II: fish-scale and cobweb type, a few focal echo density areas < 20 mm; (**d**) Grade III: echo density bands form a continuous network, multifocal echo areas > 20 mm, masses with central fibrosis.

*S. japonicum* infection was defined, in accordance with Zhou et al.^43^, as follows: a history of living in a schistosomiasis-endemic area, contact with infested water, specific *Schistosoma* serology testing, colour ultrasound, and microscopic examination of excreta (stool, urine). Visualization of parasite eggs in the stool or urine, or positive *Schistosoma* serology, were considered evidence of *S. japonicum* infection. Unique to *S. japonicum* infection is parenchymal fibrosis, a network pattern that is often described as fish scale or tortoise shell-like. Liver fibrosis was determined by ultrasound in accordance with the World Health Organization standard for *S. japonicum* infection^44^. The ultrasound imaging characteristics in our study were assessed by two specialist (with 5–10 years of experience), independently, without knowing schistosome infection status. The agreement between the two specialist was close to 85% and third specialist is required to review the assessment results when the results are inconsistent. The liver fibrosis was graded from 0 to III: (1) Grade 0: normal and thicker light spot type, normal liver sonogram or only thick liver parenchyma echo; (2) Grade I: focal echoes in the liver parenchyma are scattered without clear boundary; (3) Grade II: fish-scale and cobweb type, a few focal echo density areas < 20 mm; (4) Grade III: echo density bands form a continuous network, multifocal echo areas > 20 mm, masses with central fibrosis^45^(Fig. 3).

### Clinical evaluation and laboratory tests

The following demographic data were collected: age and gender. Venous blood samples were collected in the morning following an 8- to 12 h overnight fasting period. White blood cell count (WBC), neutrophil count (NEUT), lymphocyte count (LYMPH), eosinophil count (EO), red blood cell Count (RBC), haemoglobin (HB), platelets (PLT), were measured using an automatic hematology analyser (XE-5000, Sysmex). Alanine aminotransferase (ALT), aspartate aminotransferase (AST), total bilirubin (TB), direct bilirubin(DB),Albumin (ALB), globulin (GLB), alkaline phosphatase (ALP), glutamyl transpeptidase (GGT), blood urea nitrogen (BUN), creatinine (CRE), uric acid (UA), triglycerides (TG), total cholesterol (TC), high-density lipoprotein (HDL), low density lipoprotein (LDL), fasting blood glucose (FBG) were measured using an automatic biochemical analyzer (Beckman). Serum was prepared from the peripheral blood sample and was tested using an indirect ELISA to quantify the level of *S. japonicum* egg antigen-specific IgG antibody using a diagnostic kit from Shenzhen Huakang Biomedical Engineering Co., Ltd., China^46^. All serum samples were diluted 1:100 in sample diluent and transferred into the kit's micro-titer wells. All washing and detection steps were carried out following the manufacturer's instructions. OD (optical density) values were read at 450 nm zeroed by the reagent blank wells. All serum samples were assayed in triplicate. A positive antibody test was defined as an OD value greater than 2.1 the mean OD value of the negative control serum provided with the kit as specified by the manufacturer's instruction.

### Statistical analysis

Statistical analysis was performed using SPSS version 23 (SPSS, Chicago, IL). Continuous variables were expressed as the mean ± SD. Student’s t-test or Mann–Whitney U-test was used to evaluate differences in general characteristics and laboratory test results between participants with and without liver reticular changes or IgG. Categorical variables were expressed as counts and proportions, and evaluated using the Chi-squared test or Fisher’s exact test. Univariate and multivariate logistic regression analysis (Forward: LR) were used to investigate the factors associated with liver fibrosis reticular changes in *S. japonicum* patients. Variables with statistical significance in the univariate analysis were entered into multivariate logistic regression analysis. A *P* value < 0.05 (two-tailed) was considered statistically significant.

### Ethical approval

Ethics approval was obtained from the Ethics Committee of the third Xiangya Hospital of Central South University.

## Data Availability

The datasets used and/or analysed during the current study available from the corresponding author on reasonable request.
